# Relationship between Pre-Competition Mental State and Sport Result of Disabled Boccia Athletes

**DOI:** 10.3390/ijerph17218232

**Published:** 2020-11-07

**Authors:** Magdalena Koper, Anna Nadolska, Piotr Urbański, Maciej Wilski

**Affiliations:** Department of Adapted Physical Activity, Poznań University of Physical Education, 61-871 Poznań, Poland; nadolska@awf.poznan.pl (A.N.); piotr.u.awf@gmail.com (P.U.); mwilski@wp.pl (M.W.)

**Keywords:** boccia, elite athletes, people with an impairment, sport performance, pre-competitive mental state

## Abstract

The relationship between sport result and pre-competition mental state of 109 boccia athletes was analyzed. Mental state was described by: athletic identity, self-esteem, self-efficacy for sports, hope for success, fear of failure, anxiety, and expectancy of success. Correlation analyses were made for all four boccia classes (BC1, BC2, BC3, and BC4) and revealed that only athletic identity was associated with sport result in class BC4. Four hierarchical multiple regression models (for BC1, BC2, BC3, and BC4 boccia classes) were created, with sport result as the dependent variable. Only the BC4 model was significant and included athletic identity, anxiety, self-efficacy for sports, and expectancy of success, which explained 49% of variance in sport result. BC4 class results indicate that psychological variables have a potential impact on sport performance in boccia, and the type and level of disability should be taken into account.

## 1. Introduction

Boccia is one of the fastest growing Paralympic sports, and one of the only two ones that do not have an Olympic counterpart. It is governed by the Boccia International Sports Federation (BISFed). All events are mixed gender and feature individual, pair, and team competitions for a total of seven medal events. Boccia is a target game that requires a high degree of concentration, muscle control, accuracy, and tactical awareness [1].

Boccia is played competitively at national and international level by athletes with an impairment who require a wheelchair for locomotion. The sport was originally designed for individuals with cerebral palsy but has spread its range to include athletes with other severe disabilities that affect motor skills. For competition purposes, depending on their physical and functional abilities, boccia athletes are assigned to one of the four sports classes: BC1, BC2, BC3, or BC4 [2]. In BC1 class, athletes throw the ball with the hand or foot. They may compete with an assistant who stays outside of the competitor’s playing box. An assistant may help to stabilize and place playing chair and likewise pass the ball to the player when requested. BC2 players throw the ball with the upper limb. They are not eligible for assistance. BC3 athletes present very severe locomotor dysfunctions in all four extremities, they have no sustained grasp or release action and although they may have arm movement, they have insufficient range of movement to insert a Boccia ball onto the court. They may use an assistive device such as a ramp to pass the ball and can also exploit an assistant. Players in BC4 class have severe locomotor dysfunction of all four extremities as well as poor trunk control. Those athletes can demonstrate sufficient dexterity to throw the ball onto the court. They are not eligible for assistance [2].

The growing number of people participating in boccia on a competitive level gives rise to the need to maximize sporting performances in the most efficient way. The level of sports ability is increasing, and competition is growing, which makes the achievement of success more difficult and requires comprehensive professional sports preparation. Therefore, there is a need to conduct research among athletes practicing boccia to provide guidance for the development of effective training methods and to develop coaching models useful in practice.

Despite the development in recent years of scientific research on various aspects of creating professional conditions for para-athletes to achieve sporting excellence, the knowledge in this area is still fragmentary and incomplete, particularly in relation to developing sports such as boccia, which are working out a model of good practice in the field of professional preparation of sports players [3].

### Mental Factors and Sport Performance in Boccia

It is commonly accepted that psychological factors (e.g., mental state) are important in achieving sporting success [4,5]. Their role is increasing in the case of sport at the highest level, when the players’ skills are similar and possible success or failure is decided by the details. Existing research indicates that numerous psychological factors (relating to a positive personality, motivation, confidence, focus, and perceived social support) protect elite athletes from the potential negative effect of stressors and are thereby conducive to the attainment of optimal sport performance [6]. Although, in the last years, the body of research on sport psychology has expanded, an open question remains as to whether the findings for mental preparation also apply to athletes with disabilities. Some study findings show a lack of difference in the sport-specific psychological profiles of elite athletes with and without disabilities [7], but other research results, specific to disability sport, illuminate the subtle differences between disability and nondisability sport [8]. For example, research shows differences in the subjective resources among athletes with and without disabilities, indicating different levels of achievement motivation, sports personalities, different expectations of the effects of physical activity, etc. [9,10,11]. These findings suggest that it is not easy to transfer the results obtained among able-bodied athletes to athletes with disabilities. It should also be remembered that these differences are conditioned by athletes’ degree of commitment to sport and the level and type of disability [8]. Therefore, there is a need for knowledge of psychology in reference to the preparation of athletes with disabilities to compete in a particular sport and on a specified sport level.

It should be emphasized that there is a dearth of information on the experiences of sports people with cerebral palsy, particularly relating to the psychological preparation of boccia players [3,12]. Few existing studies outline the importance of deepening the relationship between mood and perceived performance in highly competitive environments for people with disabilities, including boccia athletes [13]. Based on the available literature and the current state of knowledge, we empirically verify the associations of several psychological variables with the obtained sport result of elite boccia players. One of the most frequently mentioned psychological constructs associated with achievement in sports is athletic identity. Athletic identity is defined as the degree to which individuals identify themselves with the role of an athlete [14]. Previous research has shown that high athletic identity has a variety of positive and negative consequences on sport involvement; however, some studies indicate that high athletic identity may be necessary for success in high level sport [15,16,17]. These studies have shown that it can be an important factor affecting the psychological variables that are directly associated with sport performance. Strong athletic identity is reflected in the way in which an individual evaluates his or her competence and worth [18,19,20], and these evaluations, like self-efficacy, self-esteem, or outcome expectancies, have been proved to have a positive relationship with task performance or sport performance [21,22,23,24,25,26,27]. According to cognitive-behavioral theory, perception of situation based on cognitive appraisal influences one’s emotional and motivational responses, which also can have an important relationship with sport performance. Among those emotional components that are considered to be key factors for high achievement, the most frequently mentioned is the anxiety level experienced by the athlete before the start. Although empirical findings investigating the anxiety-performance relationship have proved somewhat inconsistent [28,29], generally it has been suggested that anxiety level is an important factor in sport performance and outcome [30]. According to motivational response, studies suggest that achievement motivation is one of the most significant predictors of performance and is essential to participate in a competition [25]. Achievement motivation is manifested by a willingness to achieve the best possible result. The strength of motivation depends on two opposing factors: hope for success and fear of failure [31]. Lower fear of failure and more positive internal attributes regarding failure and success lead to greater persistence in sport endeavors [32,33]. According to Kämpfe et al. [11], a study on disabled athletes, hope for success especially is an important precondition of athletic achievement.

Summing up, the results of our study aim to show which of the mental factors (from psychological variables such as athletic identity, self-esteem, self-efficacy, outcome expectancies, motivation for achievements, and level of perceived anxiety) can be associated with sport results reached by disabled elite boccia athletes participating in a prestigious tournament. To the best of our knowledge, this is the first study to analyze the pre-competitive mental state of boccia athletes.

## 2. Methods

### 2.1. Participants and Procedure

Data collection was conducted by the authors at the Boccia World Open Championships in Poznan, Poland, in 2015 (WO Poznan 2015). This tournament was among the most important in the world in the BISFed calendar in 2015, with 143 participants from 31 countries competing for qualification points for the Rio 2016 Paralympics. The sampling of boccia athletes was purposive and the inclusion criteria of the study were: an active member of a national team with nominations for participation in top international competitions and active participation in the BC1, BC2, BC3, or BC4 individual events at WO Poznan 2015. The research was carried out under the auspices of the Polish Boccia Federation and BISFed. Original versions of the questionnaires used were translated into Polish, Spanish, Russian, English, and French by external experts in the field who speak both source and target languages fluently, using a bidirectional (forward and backward) translation procedure.

At the briefing for the coaches before the competition, the research project was presented; all the players taking part in the tournament were invited to participate in the study. The team leaders were given the corresponding coded questionnaires for the players and asked that they be filled in by the players before the competition began. In total, 115 completed questionnaires were returned. Because only entirely filled questionnaires were taken into consideration, questionnaires from 109 athletes were included in the statistical analysis.

### 2.2. Measures

Sport result was defined by the place the player gained in the BC1, BC2, BC3, or BC4 individual events at WO Poznan 2015. Accordingly, the smaller the number in the results (i.e., for BC1: 18, 17, 16…3,2,1 place) the better the place won, which means the better sports result achieved. Because of different numbers of competitors in particular sport classes (see Table 1), the ranking method was used in percentage terms for standardizing the score of the result obtained by the players. Therefore, sports result is given as a percentage of the place the player took in the BC1, BC2, BC3, or BC4 individual events, and the lower score means the better sport result.

The study was based on the following instruments.

1. AIMS (Athletic Identity Measurement Scale) [34]. AIMS features seven items pertaining to affective, behavioral, and cognitive aspects of identification with the athlete role (e.g., “I have many goals related to sports”). Respondents rate the extent to which they agree with each of the items on a scale from 1 (strongly disagree) to 7 (strongly agree). The total score determines the level of athletic identity, where a higher score means a stronger identification with the role of an athlete. Previous analysis has revealed its high internal consistency (*α* = 0.81 to 0.83) in studies involving nondisabled persons [14,34] and in studies involving persons with spinal cord injury (*α* = 0.90) [35]. The internal consistency rate of this scale in our study was: BC1 *α* = 0.69, BC2 *α* = 0.81, BC3 *α* = 0.89, and BC4 *α* = 0.85.
2. SES (Rosenberg Self-Esteem Scale) [36,37]. This scale is a uni-dimensional tool that allows assessment of the level of general self-worth by measuring both positive and negative feelings about the self; it consists of ten items (e.g., “On the whole, I am satisfied with myself”). The internal consistency rate of the SES in our sample was BC1 *α* = 0.70, BC2 *α* = 0.72, BC3 *α* = 0.81, and BC4 *α* = 0.69.
3. SSA scale (Self-Efficacy for Sport Activities Scale). Self-efficacy towards physical activities refers to the extent to which a person is convinced that s/he is able to stick to an exercise program even in unfavorable conditions [38]. This scale consists of 12 items, and respondents express their opinion on a seven-point scale (e.g., “I am sure I will be able to attend my planned physical activity, even when: … I’m tired”). A higher total number of points means higher self-efficacy in sport. The internal consistency rate of the SSA scale in our sample was: BC1 *α* = 0.95, BC2 *α* = 0.91, BC3 *α* = 0.88, and BC4 *α* = 0.93.
4. AMS (Achievement Motives Scale). AMS is a proven and frequently used tool in the evaluation of motivation achievements [39]. The ten-item scale consists of two independently measured components: HS: hope for success (e.g., “I like being confronted with a difficult athletic task”) and FF: fear of failure (e.g., “I find it unsettling to do something in sport, when I am not sure I can accomplish it”). Answers to individual questions are given on a four-point scale. Our study showed an acceptable internal consistency rate of the two subscales of AMS (HS: BC1 *α* = 0.84, BC2 *α* = 0.64, BC3 *α* = 0.58, and BC4 *α* = 0.73; FF: BC1 *α* = 0.76, BC2 *α* = 0.66, BC3 *α* = 0.62, and BC4 *α* = 0.80).
5. STAI (State Trait Anxiety Inventory). STAI is a tool designed for studying anxiety understood as a transitional and situational determined state of an individual and fear understood as a relatively stable personality trait [40]. In our study, only one of two subscales was used, measuring anxiety as a state. It consists of 20 statements (e.g., “I feel upset”), to which the tested person refers using a four-point scale. Cronbach’s Alpha in our sample was: BC1 *α* = 0.87, BC2 *α* = 0.77, BC3 *α* = 0.84, and BC4 *α* = 0.62.
6. Expectancy Scale. STPQ (Self- and Task-Perception Questionnaire) contains 19 questions concerning beliefs, attitudes, and values towards the expected accomplishments [41]. For the purpose of the study, only five questions from the expectancy subscale were used (e.g., “How good at boccia are you?”). Cronbach’s Alpha in our study was: BC1 *α* = 0.91, BC2 *α* = 0.94, BC3 *α* = 0.87, and BC4 *α* = 0.92.

A demographic questionnaire to gather personal data, such as date of birth, sex, and sports experience, was also distributed to the contestants.

### 2.3. Data Analysis

Statistical data analysis was performed using STATISTICA 13.0 data analysis software system (TIBCO Software Inc., 2017, Tulsa, OK, USA). Cronbach’s Alpha coefficient (*α*) was calculated to measure internal consistency of the scales used in our research. The Shapiro–Wilk test was used to check for normal distribution of the data. Analysis of variance (ANOVA) were performed to determine if there are statistically significant differences between sports classes on independent variables (all the psychological variables: athletic identity, self-esteem, self-efficacy for sport, hope for success, fear of failure, anxiety, and expectation of success). The ANOVA was used for variables with a normal distribution and homogeneous variations. If the normality distribution of the variables was not met for at least one of the tested sports classes, the nonparametric Kruskal–Wallis H test (analysis of variance on ranks) was used, and when the test was significant, the Kruskal–Wallis post hoc test for multiple comparisons would be performed. To describe differences related to sport classes on psychological variables, the effect sizes (Cohen’s d coefficient) were calculated as the difference between means divided by the within standard deviation of the difference [42]. Using Cohen’s criteria, an effect size ≥ 0.20 and < 0.50 was considered small, ≥ 0.50 and < 0.80 medium, and ≥ 0.80 large. Then the data were screened and checked against the assumptions of regression analysis. Pearson’s correlation coefficient and t-test were used to analyze the relationships between the independent variables and sports result achieved by the boccia players for each sport classes. Finally, we performed four separate hierarchical multiple regression models (respectively for all classes) with the achieved sport result as the dependent variable and all the mental factors as independent variables. The level of statistical significance was set at *p* < 0.05.

## 3. Results

### 3.1. Group Characteristics

The sample comprised a total of 109 boccia athletes (77% of all contestants) representing 27 countries participating in the tournament. The study group included 24 (22%) women and 85 (78%) men, aged between 16 and 54 years (*M*_age_ = 29.8 years, *SD*_age_ = 9.3). Study participants were athletes representing all sport classes that apply in boccia (Table 1).

### 3.2. Psychological Correlates of Sport Result in Boccia Athletes 

Before testing the hypotheses regarding the association between psychological factors and sport result in boccia athletes, we investigated the relationships between sex, sport experience, and sport result to examine whether we should control for these variables in future analyses. The data showed that sex (male: 0.46 ± 0.29, female: 0.54 ± 0.28; *t* = 1.27, *p* = 0.205) and sport experience (*r* = −0.17, *p* = 0.080) were unrelated to sport result when calculated on all the athletes participating in our study, as well as for each sports classes respectively (in all cases *p* ≥ 0.05). We also examined whether players from different sport classes differ in terms of psychological variables (Table 2). Only for the anxiety variable there were significant differences in the mean values. Anxiety level was significantly higher in BC4 than BC2 group (*p* = 0.043, *d* = 0.92) and between BC4 than BC3 group (*p* = 0.004, *d* = 0.91).

Table 3 depicts the correlations between psychological variables and sport results achieved by the boccia players for each sport class.

Only one factor—namely athletic identity—was significantly associated with sport results achieved by the athletes. Analyzing the results of BC1, BC2, and BC3 class competitors, there were no significant associations between the mentioned variables. In the case of BC4 athletes, a higher level of athletic identity was associated with a poor sport result achieved (a higher variable’s score). Despite such results, an attempt was made to create four separate hierarchical multiple regression models (for all classes) with sports score achieved by the athletes as the dependent variable. Multivariate analyses have the advantage of accounting for intercorrelations between the various predictor variables and identifying variables that make a unique contribution to the prediction of sport results. After all, only the BC4 model was significant and explained a considerable proportion of the variance in sports results achieved by the athletes (Table 4). According to the BC4 model, sports results were predicted by four variables, two of which were significant (*p* < 0.05): athletic identity and anxiety.

## 4. Discussion

The principal aim of this study was to analyze the relationship between sport result and pre-competition mental state of boccia players. The main assumption of our study was that the level of athletic identity of boccia elite players has an important impact on cognitive appraisals, which include self-efficacy, self-esteem, and outcome expectancies presented by the contestant before the start, and it is posited that these influence emotional and motivational responses. All these psychological variables together affect actual sporting performance. Based on this theoretical assumption, correlation analyses were made for all four classes and revealed that athletic identity was associated only with sport result in class BC4. Our studies did not show any other relationships. Additionally, we performed multivariate regression analysis for all four classes. As a result, only the BC4 model was significant. Specifically, we demonstrated that BC4 athletes presenting with higher levels of self-efficacy and outcome expectancies and lower levels of athletic identity and anxiety before the start achieved better sport results. A regression model with all independent variables explained up to 49% of variance in the dependent variable. The question of why we found correlates in only one group remains open. Perhaps the disability factor plays a more important role than we originally expected.

It should be noted that although boccia was originally designed for people with cerebral palsy, it is currently available for athletes with other severe impairments. According to the applicable regulations, BISFed provides an opportunity to compete in boccia not only for individuals with severe neurological impairment affecting the central nervous system (CNS) (including spastic hypertonia, dystonia, athetosis, and ataxia in all four limbs) but also for individuals with severe locomotor dysfunction in all four limbs of noncerebral origin (such as muscular skeletal disorders and limb deformities) [2]. While classes BC1, BC2, and BC3 are classified as players with cerebral palsy, BC4 is only for athletes who are diagnosed with conditions of noncerebral origin. Our results may suggest that in the case of athletes with cerebral palsy, sport result depends on factors other than we expected. Cerebral palsy can essentially affect any body function or structure dependent upon its severity and type but is most typically associated with limitations in gross motor functioning, muscle spasticity, and sometimes with cognitive impairment [43]. Boccia is dedicated to people with physical disabilities and during the classification process intellectual limitations are not verified. Therefore, it is difficult to determine whether and to what extent cognitive limitations potentially associated with severe CNS impairment may have influenced (1) understanding and interpretation of the statements contained in the questionnaires; and (2) characteristics of the pre-competition mental state and its predictive role in the sport result. Nonetheless, it points to the need for a specific approach to the mental preparation of this group of athletes.

The results of the BC4 class are only partially in line with our assumptions. Primarily, we assumed that higher levels of athletic identity would promote sport performance. The result turned out to be the opposite, which undermines the results of research suggesting that athletic identity promotes sporting achievement [16,17]. However, in the case of our group, we are dealing with elite athletes competing at the championship level. According to Tasiemski et al. [44], identification with the athletic role becomes more salient with the level of sport participation. Brewer et al. [14] have argued that there are potential risks associated with over-identification with the athletic role. Too strong identification generates emotional tension in the starting situation, which interferes with achieving the best results. It is likely that in the case of elite athletes, athletic identity ceases to play a stimulating role and, conversely, may adversely affect sport performance, as evidenced by the results of our research.

This assumption is confirmed with broader analysis of our regression results. A lower level of athletic identity was associated with a lower level of precompetitive anxiety and better sport performance in the BC4 group. It has been proved that high levels of anxiety influence the quality of performance, because they increase muscle tension, undermine coordination, and destroy proper focus and attention direction [7]. These properties are especially needed in boccia, which requires the coordination of extremities and emotional control [45]. However, a growing body of literature suggests that state anxiety has both a facilitative and a debilitative function for performance [46,47]. According to Hanton et al. [28], one of the factors regulating this function is self-efficacy, which is a belief in one’s ability to produce a particular level of performance [48]. Meta-analysis by Moritz et al. [21] provides clear evidence of a significant relationship between self-efficacy and performance. Based on social cognitive theory [48], anxiety is typically a function of an individual’s level of confidence in performing a given activity and increases in self-efficacy, acting as a mechanism for anxiety reduction [30]. This assumption is supported by our results, indicating that a lower level of anxiety was associated with a high level of self-efficacy in boccia BC4 players. Together, these variables proved to have a positive influence on performance, which is in line with earlier studies indicating that high self-efficacy is associated with low anxiety and contributes to better performance in competition [22,30,49]. It is also worth noting that in the BC4 group we observed the highest level of precompetitive anxiety (in comparison to other classes), which may also be important in the context of the results analysis.

The last variable associated with sport result in our study was outcome expectancy. Positive outcome expectancies are beliefs about self, or what individuals view as their probability for success at a specific task [50]. Such beliefs flow from self-efficacy and directly impact behavior [51]. In our study, athletes from the BC4 group who had positive outcome expectancies achieved a better sport result. This finding is in line with earlier studies indicating that expectancy beliefs are highly correlated with actual outcomes or performance [24,52,53].

A few words should be devoted to the results for other variables. Self-esteem and motivation for achievements were not included in any of the regression models, which is in opposition to the data published by other authors [25,27,32,33]. Perhaps these variables are of minor importance for athletes with disabilities; however, this assumption requires a much broader comparative research.

Our research does not allow for the presentation of specific practical implications but provides knew knowledge, which may serve as a basis for further research. In particular, two issues should be taken into account. The first concerns the disability and its impact on psychological variables. Our research has shown that disability is a differentiating factor for the group and impedes research. When we talk about athletes with disabilities, we have to remember that disability is a powerful factor that often transcends other psychological variables [54]. This particularly applies to such types of disability as cerebral palsy, which are characterized by great diversity and the probability of cognitive impairment. There is a high degree of inter-individual variability affected by level and type of disability, which complicates the diagnosis of cognitive–emotional factors associated with successful and poor sport performance. In the preparation of athletes practicing boccia, coaches or sport psychologists should ensure the pre-competitive mental state of an athlete, taking into account the individual differences between players in each sport class. Most likely it will require an even more individualized approach. The second area of future research indicates associations identified in the BC4 group. It is certainly worth analyzing more accurately the relationship between pre-competition cognitive variables and sport performance, while cognitive appraisals are potentially modifiable. The results of this study suggest that intervention aimed at the improvement of self-efficacy and positive outcome expectancies may reduce the level of pre-competition anxiety and therefore promote better sport performance in disabled athletes.

Several limitations of the current study should be mentioned. Firstly, our analysis was based on self-reported data, which might constitute a source of bias. Moreover, we used a one-shot questionnaire survey, which always poses a risk for result falsification due to a tendency to respond uni-directionally. Secondly, results were based on a cross-sectional survey; therefore, causal relationships between variables could not be inferred. Our assumption about the impact of the analyzed variables on sport performance was driven by the results of previous studies conducted with different populations and the current state of knowledge. Thirdly, our findings should be interpreted with caution because of the small sample size. However, research on elite athletes will necessarily imply small sample sizes and the inclusion of 109 athletes at the elite level should be considered a strength of the present study. We are aware that statistical analyses were made on smaller samples, and hierarchical regression models are not regarded as being particularly powerful. This is one of the strongest limitations, not allowing us to draw constructive conclusions, but we saw this action as legitimate because of the unique specificity of the study group.

Fourthly, the results presented here should be generalized with caution because of the small representation of women (22%). This study does not verify the importance of gender in the context of the research. Finally, the athletes were assessed during one sporting event and their level of performance could also depend on their temporary psychophysical health disposition or other uncontrolled factors, especially skill level.

## 5. Conclusions

In conclusion, we failed to identify the psychological variables that contribute to sport performance in boccia players. We assume that the cause was the strong influence of the disability factor. The type and level of disability should be taken into account when designing psychological interventions aimed at optimum preparation of boccia athletes to compete. However, our results also indicate that psychological variables have a potential positive impact on the sport performance of disabled athletes—in particular, when it comes to athletic identity, cognitive variables such as self-efficacy and outcome expectancy, and related emotional variables such as pre-competition anxiety level—which sets the direction for future research.

## Figures and Tables

**Table 1:** Characteristics of boccia athletes by class sports.

Sport Classes in Boccia		BC1	BC2	BC3	BC4
Research participants (frequency) *		75% (18)	74% (25)	88% (42)	69% (24)
Sex:	Male (frequency)	78% (14)	80% (20)	74% (31)	83% (20)
	Female (frequency)	22% (4)	20% (5)	26% (11)	17% (4)
Age at time of study (*M* ± *SD*)		33.9 ± 11.1	28.1 ± 7.9	29.0 ± 8.8	29.9 ± 9.8

* Percentage of respondents among the contestants in each sport class (representing Australia, Belgium, Bermuda, Canada, Chile, Czech Republic, Denmark, France, Germany, Great Britain, Greece, Ireland, Israel, Japan, Macau China, Mexico, Norway, Poland, Portugal, Russia, Slovakia, Slovenia, Spain, Sweden, Taipei China, Turkey, and United States).

**Table 2:** Differences in psychological variables between boccia athletes by sport classes (BC1, BC2, BC3, and BC4).

Psychological Variables	BC1 *n* = 18	BC2 *n* = 25	BC3 *n* = 42	BC4 *n* = 24
	*M* ± *SD*			
Athletic identity ^b^	37.78 ± 7.13	38.00 ± 7.64	36.74 ± 9.86	39.25 ± 7.55
Self-esteem ^a^	27.83 ± 5.78	30.00 ± 4.86	30.52 ± 6.34	28.54 ± 5.30
Self-efficacy for sports ^b^	53.11 ± 19.19	54.48 ± 17.86	57.98 ± 15.56	49.17 ± 17.34
Hope for success ^b^	10.78 ± 4.52	11.60 ± 3.21	10.83 ± 3.23	9.08 ± 3.63
Fear of failure ^b^	5.56 ± 3.35	6.00 ± 3.18	5.19 ± 3.14	6.17 ± 3.70
Anxiety ^b^	38.67 ± 9.74	37.60 ± 7.53	36.86 ± 9.16	44.17 ± 6.74 *
Expectation of success ^b^	25.39 ± 5.66	25.64 ± 5.55	25.40 ± 5.19	26.42 ± 6.09

^a^—ANOVA; ^b^—Kruskal–Wallis H test; *—significantly different from BC2 and BC3.

**Table 3:** Relationship between psychological variables and sport results among boccia athletes (BA) by sport classes (BC1, BC2, BC3, and BC4), expressed by correlation coefficient.

	Sport Results Among BA			
Psychological Variables	BC1	BC2	BC3	BC4
Athletic Identity	0.08	−0.12	0.05	**0.44**
Self-Esteem	0.45	−0.09	0.28	−0.31
Self-Efficacy for Sports	−0.37	−0.04	0.14	−0.33
Hope for Success	−0.26	0.38	−0.03	−0.20
Fear of Failure	0.28	0.01	−0.05	0.31
Anxiety	0.25	−0.10	0.02	0.39
Expectation of Success	0.03	0.03	0.21	−0.25

Correlations significant at *p* < 0.05 are marked in bold.

**Table 4:** Regression analysis predicting sport results among boccia players in BC4.

Variables	*R ^2^*	*β*	*F*	*p* Value
Model BC4	0.49		4.48	**0.0102**
Athletic Identity		0.016		**0.025**
Anxiety		0.018		**0.022**
Self-Efficacy for Sports		−0.005		0.127
Expectation of Success		−0.011		0.165

Significant *p*-values are marked in bold.
